# Economic evaluation of advanced practice physiotherapy models of care: a systematic review with meta-analyses

**DOI:** 10.1186/s12913-021-07221-6

**Published:** 2021-11-09

**Authors:** Simon Lafrance, Anthony Demont, Kednapa Thavorn, Julio Fernandes, Carlo Santaguida, François Desmeules

**Affiliations:** 1 School of Rehabilitation, Faculty of Medicine, Université de Montréal, Montreal, Quebec Canada; 2 Maisonneuve-Rosemont Hospital Research Center, Université de Montréal Affiliated Research Center, Montreal, Quebec Canada; 3 INSERM 1123 ECEVE, Faculty of Medicine, Paris-Diderot University, Paris, France; 4 Physiotherapy School, University of Orleans, Orleans, France; 5 School of Epidemiology and Public Health, University of Ottawa, Ottawa, ON Canada; 6 Ottawa Hospital Research Institute, Ottawa, ON Canada; 7 Hôpital du Sacré-Coeur de Montréal Research Center, Université de Montréal Affiliated Research Center, Montreal, Quebec Canada; 8 Department of Surgery, Faculty of Medecine, Université de Montréal, Montreal, Quebec Canada; 9 Department of Neurology and Neurosurgery, Faculty of Medecine, McGill University Health Center, Montreal, Quebec Canada

**Keywords:** Physical therapy specialty, Physical therapists, Health planning, Economics, Cost analysis; health care costs, Health expenditures, “Physiotherapy”, “Physical therapy” and “advanced practice”

## Abstract

**Background:**

The objective of this systematic review is to appraise evidence on the economic evaluations of advanced practice physiotherapy (APP) care compared to usual medical care.

**Methods:**

Systematic searches were conducted up to September 2021 in selected electronic bibliographical databases. Economic evaluation studies on an APP model of care were included. Economic data such as health care costs, patient costs, productivity losses were extracted. Methodological quality of included studies was assessed with the Effective Public Health Practice Project tool and the Critical Appraisal Skills Programme checklist. Meta-analyses were performed and mean differences (MD) in costs per patient were calculated using random-effect inverse variance models. Certainty of the evidence was assessed with the GRADE Approach.

**Results:**

Twelve studies (*n* = 14,649 participants) including four randomized controlled trials, seven analytical cohort studies and one economic modeling study were included. The clinical settings of APP models of care included primary, emergency and specialized secondary care such as orthopaedics, paediatrics and gynaecology. The majority of the included participants were adults with musculoskeletal disorders (*n* = 12,915). Based on low quality evidence, health system costs including salaries, diagnostic tests, medications, and follow-up visits were significantly lower with APP care than with usual medical care, at 2 to 12-month follow-up (MD: -139.08 €/patient; 95%CI: -265.93 to -12.23; *n* = 7648). Based on low quality evidence, patient costs including travel and paid medication prescriptions, or treatments were significantly higher with APP care compared to usual medical care, at 2 to 6-month follow-up (MD: 29.24 €/patient; 95%CI: 0.53 to 57.95 *n* = 1485). Based on very low quality evidence, no significant differences in productivity losses per patient were reported between both types of care (MD: 590 €/patient; 95%CI: -100 to 1280; *n* = 819).

**Conclusions:**

This is the first systematic review and meta-analysis on the economic evaluation of APP models of care. Low quality evidence suggests that APP care might result in lower health care costs, but higher patient costs compared to usual medical care. Costs differences may vary depending on various factors such as the cost methodology used and on the clinical setting. More evidence is needed to evaluate cost benefits of APP models of care.

**Supplementary Information:**

The online version contains supplementary material available at 10.1186/s12913-021-07221-6.

## Background

Health care expenditures have been drastically increasing over several decades. In the last 50 years, the proportion of the gross domestic product dedicated to health care has almost doubled among Organisation for Economic Co-operation and Development (OECD) countries [1]. Physical disorders, leading to pain and disability represent an important proportion of health care costs, with annual expenditure up to USD 635 billion for the treatment of pain in the United States [2–4]. Despite this increase in health care spending, timely access to care remains problematic [5, 6]. Physicians have increasing difficulty meeting this growing demand and simply increasing the number of physicians does not appear to be an efficient solution, especially in the long term [5–7]. Health care delivery transformations are warranted in order to offer more efficient care to a growing number of patients [8, 9]. Expanding and extending clinical roles of allied health care providers appears to be a promising solution to manage this growing demand [10, 11].

For acute and chronic physical disorders, the use of advanced practice physiotherapy (APP) models of care (MoC) has been proposed as a potential solution to improve health care access [12–17]. The ultimate goals of these MoC are to provide best practice care while improving health care access in a cost-effective manner. APP MoC may include role enhancements and role substitution for physiotherapists related to traditionally performed medical or controlled acts, such as diagnosis, ordering imaging or laboratory tests, triaging potential surgical candidates and referring of patients to other medical specialists [13, 18]. APP MoC can be developed and implemented in various clinical settings such as in primary, emergency and secondary care. In these models, more complex cases requiring medical assessment or potential surgical candidates are referred to medical doctors while less complex patients can be managed independently by advanced practice physiotherapists (APPs).

Previous systematic reviews report that APP MoC improves access to care by reducing waiting time for an initial consultation while providing at least comparable quality of care and retaining high patient satisfaction for adults with musculoskeletal disorders (MSKDs) [12–15, 17, 19]. Three systematic reviews, evaluating several outcomes and not only economic evaluations of APP MoC have been published. The first review was published nearly a decade ago [13]. One review was limited to studies in emergency departments [14], and the other one included studies in primary and secondary care [19]. Based on two trials in emergency care, Matifat et al. [14] reported no significant differences in terms of health care costs between APP care and usual medical care (UMC) and Marks et al. [19] reported that APP care may be cost saving based on one trial in orthopaedic care. None of these performed a recent comprehensive review of all studies on the economic evaluation of APP MoC and neither performed a meta-analysis. Therefore, the aim of this systematic review is to summarize and appraise the available evidence on the economic evaluations of APP MoC in primary, emergency, and secondary care in terms of health care system costs, patient costs and productivity losses.

## Methods

This review protocol is available online on Prospero (https://www.crd.york.ac.uk/prospero/). The registration number is: CRD42020185050. There were no amendments to the protocol.

### Literature search

Bibliographical searches were conducted using four electronic databases (Medline, Embase, Cochrane Central and CINAHL) from their inception to September 2021, using terms related to advanced practice, physiotherapy and economic evaluation (full search strategy in supplementary materials). The reference lists of identified published studies and of previous relevant systematic reviews were screened for any additional eligible studies.

### Study selection

Two reviewers (SL and AD) independently reviewed titles and abstracts to identify relevant studies. Consensus of the two reviewers was needed to include the studies. A third reviewer (FD) was available if consensus was not achieved by the two initial reviewers. Articles were included if they met the following inclusion criteria: 1- included the evaluation of an APP MoC; APP was defined as a role involving a higher level of practice and responsibilities for physiotherapists including more complex clinical responsibilities, role enhancement and medical role substitution with or without the addition of delegated medical or controlled acts [18]; 2- presented any type of economic evaluation including cost-minimization, cost-effectiveness, cost-utility or cost-benefit analyses and from any economic perspective (health care system, patient or societal); 3- enrolled patients were cared by an APP, and 4- articles were written in French or English. Studies including patients with cancer-related pain, degenerative neurological disorder and/or autoimmune disorders were excluded.

### Data extraction

Data of included studies were extracted with a predefined standardized form that documented study design, population, study setting, number of participants, patients diagnoses and characteristics, types of APP MoC and UMC, the costs measured (health care system costs, patient costs and productivity losses), types of economic evaluations (cost-minimization, cost-effectiveness, cost-utility or cost-benefit analyses) and length of the follow-up. Cost-minimization represents an analysis of the difference in costs between APP care and UMC when the clinical effectiveness of the two approaches is considered equivalent. Data extraction was performed by one evaluator (SL) and verified by a second evaluator (AD). When data were missing or incomplete, original authors were contacted to obtain complete data and results.

### Methodological quality assessment

The methodological quality of included studies was assessed with the Effective Public Health Practice Project (EPHPP) tool (available at http://www.ephpp.ca/tools.html). The EPHPP tool is a generic appraisal tool developed for use in public health research. The tool can evaluate any study design and it appraises selection bias, study design, confounding, blinding, data collection method and number of withdrawals/dropouts. The EPHPP tool has appropriate content and construct validity [20] as well as good intra- and inter-rater reliability [20, 21]. Since the EPHPP tool is not specific to economic studies, sections A and B of the Critical Appraisal Skills Programme (CASP) checklist developed by the Public Health Resource Unit, was also used in order to assess specifically the quality of the economic analyses [22]. The CASP checklist only evaluates the methodological quality of the economic component of a study and does not evaluate other methodological biases such as selection bias and study design, confounding, blinding or number of withdrawals/dropouts. The assessment of the methodological quality using these two complementary tools was performed by two independent evaluators (SL and AD); final scores were obtained through a consensus. In case of disagreement, a third reviewer was available to facilitate consensus (FD). Sources of funding were also verified.

### Data synthesis

All costs were adjusted for inflation according to the study’s original country and then converted to euro for the 2020 fiscal year based on the Bank of England’s inflation and conversion rates, since the majority of the original studies calculated costs to euro [23]. Rates used for conversion and inflation adjustment are available in supplementary material.

Randomized controlled trials (RCTs) and cohort studies that measured costs from similar perspectives, such as the costs from the health care system, patients’ costs or productivity losses were pooled together into separate meta-analyses through Review Manager (RevMan 5.4, The Cochrane Collaboration, Copenhagen, Denmark). Meta-analyses were also calculated according to the clinical settings (primary, emergency, orthopaedic or paediatric orthopaedic care). Two secondary meta-analyses were conducted: one including only RCTs and one including studies comparing APP MoC to nurse practitioners care (and not compared to UMC). Mean differences (MD) in costs were calculated. The inverse variance method was used to weigh each study and was calculated using random effect models, as it “provide a result that may be viewed as an average intervention effect” [24]. Missing standard deviations were calculated using the RevMan Calculator (available at https://training.cochrane.org/resource/revman-calculator). Alpha level was set at 0.05. For studies not pooled into meta-analyses, a narrative synthesis was performed.

The GRADE Approach (Grading of Recommendations, Assessment, Development and Evaluations) was used to grade the overall quality of the evidence. For results based only on RCTs certainty was initially considered as high while pooled results from RCTs and observational studies combined were considered as moderate. Thereafter, certainty could be rated down based on factors such as risk of bias, imprecision, inconsistency, indirectness and potential publication bias while it could be rated up if a large magnitude of effect was observed [25, 26].

## Results

**Fig. 1:**
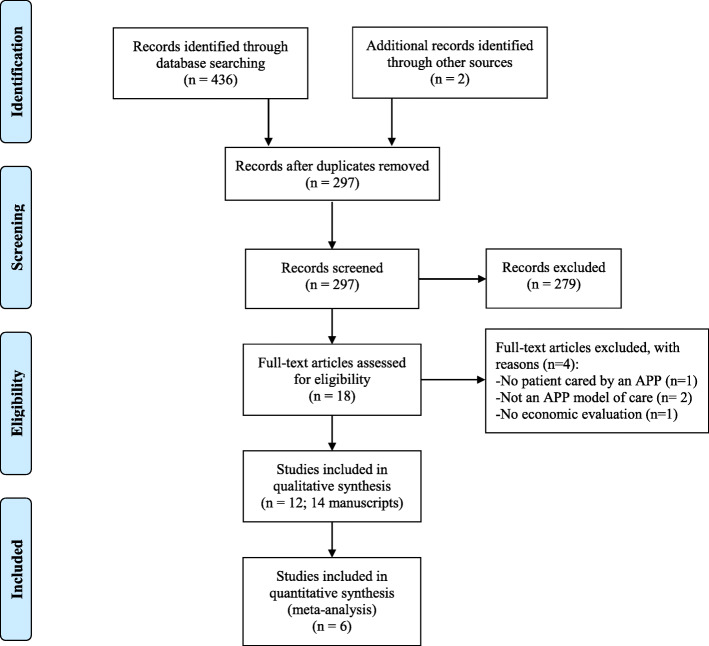
Schematic breakdown of literature search results

From the 18 potentially relevant articles identified through titles and abstract review, 12 studies (*n* = 14,649 participants; 14 articles) met the eligibility criteria after full-text review (Fig. 1). Detailed characteristics of included studies are presented in Table 1. Studies were excluded because they include no patient cared by an APP [27], did not study an APP MoC [28, 29] or did not include economic data [30].

**Table 1:** Characteristics of included studies (*n* = 12)

Study Design	Authors year	Country	Setting	Patients Characteristics			Advanced Practice Models of Care Characteristics			
				Sample (n)	Age (Mean years ± SD)	Gender (female, %)	APPs Experience	APPs Training	APPs role	Medical Delegated Acts
RCTs	Bornhöft et al., 2019	Sweden	Primary care	MSKDs (*n* = 55)	39.1	60%	Variable depending on the APPs	Not reported	Autonomous assessment & management	None
	Daker-White et al., 1999	United Kingdom	Orthopaedic care	MSKDs (*n* = 481)	48.5	52%	Not reported	X-ray prescription	Autonomous assessment, management & referral to medical specialists	Diagnostic imaging (X-ray, MRI), EMG & Blood tests
	McClellan et al., 2013	United Kingdom	Emergency care	Peripheral MSK injury (no fracture) (*n* = 372^a^)	85% between 17 and 44	44%	Not reported	Not reported	Autonomous assessment & management (initial triage by nurses)	Not reported
	Richardson et al., 2005	United Kingdom	Emergency care	MSK injury (no fracture) (*n* = 766)	39.3 ± 16.2	42%	Unclear	Not reported	Autonomous assessment, management & referral to medical specialists (initial triage by nurses)	Diagnostic imaging (X-ray)
Observational studies^b^	Belthur et al.,2003	United Kingdom	Paediatric orthopaedic care	Paediatric MSKDs (*n* = 932)	7.5	47%	Not reported	Residency type training in the paediatric orthopaedic unit	Autonomous assessment, management & referral to medical specialists	Not reported
	Brennen et al., 2019	Australia	Gynaecology, urogynaecology and urology care	Pelvic floor disorders (*n* = 268)	Not reported	100%	Not reported (60 APPs in total)	APP pelvic floor training	Autonomous assessment, management & referral to medical specialists	Urodynamic investigation
	Cottrell et al., 2019	Australia	Orthopaedic care	MSKDs (*n* = 44)	50.9 ± 12.4	70%	Not reported	Not reported	Autonomous assessment, management (telehealth and face-to face) & referral to medical specialists	Diagnostic imaging (X-ray, MRI & CT), blood test, injection ^c^
	Harding et al., 2018	Australia	Orthopaedic care	Hip & knee arthroplasty follow-up (*n* = 2057)	Not reported	Not reported	> 7 years in MSK care	Four days APP training & postgraduate master’s degree in MSK physiotherapy	Autonomous assessment, management & referral to medical specialists	Diagnostic imaging (X-ray, MRI & CT), blood test, injection^c^
	McGill, 2017 & McGill et al., 2021	USA	Primary care (military)	MSKDs (*n* = 8053^a^)	35.4 ± 12.7	Not reported	Not reported	10–12 days Military APP training	Autonomous assessment, management & referral to medical specialists	Diagnostic imaging & medications
	Ó Mír et al., 2019	Ireland	Paediatric orthopaedic care	Paediatric MSKDs (*n* = 534)	7.8	52%	Not reported	One-month residency type training in the paediatric orthopaedic unit	Autonomous assessment, management & referral to medical specialists	Diagnostic imaging (X-ray, MRI) & Blood tests^c^
	Peterson et al., 2021	Sweden	Primary care	MSKDs requiring X-Rays (*n* = 107)	Not reported	Not reported	> 3 years in primary care	One-Day training on X-ray prescription	Autonomous assessment, management & referral to medical specialists	Diagnostic imaging (X-ray)
Modeling ^d^	Coman et al., 2014 & Standfield et al., 2016	Australia	Orthopaedic care	MSKDs (*n* = 980)	56.7 ± 13.9	56%	Most had > 10 years	Not reported	Autonomous assessment, management (multidisciplinary team) & referral to orthopaedic specialist wait list	Diagnostic imaging (X-ray) & referrals for injections

*APP* Advanced practice physiotherapy, *APPs* Advanced practice physiotherapists, *CT* Computerized tomography, *EMG* Electromyography, *MoC* Model of care, *MRI* Magnetic resonance imaging, *MSK* Musculoskeletal, *MSKDs* Musculoskeletal disorders, *RCTs* Randomized control trials, *SD* Standard deviation, *X-ray* Radiograph

^a^Also include nurse practitioners. The study by McGill, 2017 also includes osteopathic physicians and physician assistant

^b^All observational studies are prospective, except the retrospective study by McGill, 2017

^c^Prescription of medical delegated acts through medical directives, except for X-ray in the study by Harding et al., 2018

^d^The economic modeling study included a Markov model (Coman et al., 2014) and discrete event simulation with dynamic queuing (Standfield et al., 2016)

### Study design and types of economic evaluations

Four RCTs [31–34], three prospective cohort studies [35–37], four retrospective cohort studies [38–42] and one economic modeling study (2 articles) [43, 44] were included.

All included studies measured health care system costs, five studies measured patient costs [32–35, 37] and two studies measured patients’ productivity losses [31, 34]. Four observational studies only included health care practitioner’s salary in their health care system costs outcomes (Table 3).

Cost-minimization analyses were performed in all included studies except one modeling study. A cost-utility analysis and a cost-benefit analysis were performed in one RCT [31]. One modeling study used a Markov model analysis in one manuscript [43] and a discrete event simulation with dynamic queuing [44] in another paper to assess the cost-utility of APP MoC.

APP care was compared to care provided by orthopaedic surgeons [32, 35, 36, 38, 43, 44], emergency physicians [33, 34] family physicians [31, 37, 40, 41] or gynecologists [39] . Two studies also compared APP care to nurse practitioners care [33, 40, 41] while one study also compared APP care to osteopathic physicians and physician assistant care [40, 41]. One study compared APP telehealth care to APP face-to-face care [42].

### Clinical settings and participants

Three studies were performed in primary care clinics [31, 37, 40, 41], two in emergency departments [33, 34] and seven in specialized care including four in adults orthopaedic outpatient clinics [32, 35, 42–44], two in paediatrics orthopaedic outpatient clinics [36, 38] and one in a gynaecology and urology outpatient clinic [39]. All included studies were conducted in Western Countries. More specifically, four studies were conducted in the United Kingdom [32–34, 38], four in Australia [35, 39, 42–44], two in Sweden [31, 37], one in Ireland [31, 36] and one in the United States [40, 41].

A total of 14,649 participants were included and participants were adults with MSKDs (*n* = 12,915) or pelvic floor disorders (*n* = 268) or infant or children with MSKDs (*n* = 1466). Female gender accounted for 53% of the included participants (*n* = 2334/4432). Participants in the included studies were new patients referred for an initial APP consultation in all studies except in the study by Harding et al. [35] which included follow-up of hip or knee arthroplasty patients. Overall, there was no significant difference in baseline pain, disability, and quality of life among participants assigned to APP care or UMC in RCTs as described in Table S2 in supplementary materials.

### Types of advanced practice physiotherapy models of care

In all included studies, APPs autonomously assessed, managed and referred patients to medical specialists when relevant, except in the RCTs by Bornhöft et al. [31] and by McClellan et al. [33] in which it is unclear whether APPs were able to make direct referrals to medical specialists. APPs could prescribe diagnostic imaging tests such as plain radiographs or MRI in nine studies [32, 34–37, 40–44], blood tests in three studies [32, 35, 36], nerve conduction studies in one study [32], urodynamic investigation in one study [39] and certain medications such as nonsteroidal anti-inflammatory drugs in one study [40, 41]. APPs could refer patients for corticosteroid injections in one study [43, 44]. None of the APP MoC systematically provided a comprehensive rehabilitation intervention to participants and most MoC did not specify the detail of the conservative care offered [31, 33, 34, 38, 40, 41, 43, 44]. In four MoC, APPs could refer patients to outpatient physiotherapy [35, 36, 39] or provide education and prescribe a self-management exercise program to patients in one study [32]. Full details are presented in Table 1.

### Quality of the included studies

Based on the EPHPP tool, one study was considered of high quality [31], six of moderate quality [32–35, 37, 40, 41] and five of low quality [36, 38, 39, 42–44]. Presence of confounders was unclear in all observational studies, except for the study by Belthur et al. [38] in which a confusion bias was present as patients with more severe conditions and more likely to require complex care were only seen by medical doctors. Blinding of the outcome assessors and/or participants was unclear in all observational studies and in one RCT [34]; outcome assessors, but not participants were blinded in two RCTs [31, 33] and both outcomes assessors and participants were not blinded in one RCT [32].

Based on the CASP checklist, the effects of the interventions were measured appropriately only in the four RCTs and in the modeling study [31–34, 43, 44]. Costs were properly measured and included all important resources in four studies [31, 32, 40, 41, 43, 44]. Incremental analyses were performed in two studies [31, 43, 44]. Sensitivity analyses were performed in four studies [33, 42–44]. Details are presented in Table 2.

**Table 2:**
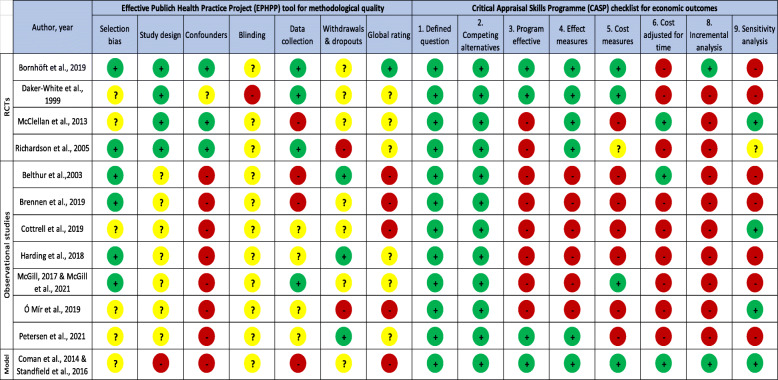
Methodological Quality of included studies based on the Effective Public Health Practice Project Tool and on the Critical Appraisal Skills Programme (CASP) checklist for economic the economic analyses component (*n* = 12)

EPHPP: Green = Strong; Yellow = Moderate; Red = Weak

CASP: Green = Yes; Yellow = Unclear; Red = No

*RCTs* Randomized controlled trials

Model: Economic Modeling analyses

†: A “no” was provided when clinical effectiveness was not directly assessed in the study but demonstrated in previous studies

‡: Costs were not adjusted in most studies, but follow-up periods were short (less than 12 months, except in McGill, 2017)

CASP checklist: 1. Was a well-defined question posed? 2. Was a comprehensive description of the competing alternatives given? 3. Does the paper provide evidence that the program would be effective? (i.e., would the program do more good than harm?) 4. Were the effects of the intervention identified, measured and valued appropriately? 5. Were all important and relevant resources required, and health outcome costs for each alternative identified, measured in appropriate units and valued credibly? 6. Were costs and consequences adjusted for different times at which they occurred (discounting)? 7. What were the results of the evaluation? (See result section) 8. Was an incremental analysis of the consequences and cost of alternatives performed? 9. Was an adequate sensitivity analysis performed?

### Economic evaluation of advanced practice physiotherapy care compared to usual medical care

#### Cost-minimization analyses

Four RCTs and six observational studies performed cost-minimization analyses. The four RCTs reported that the two MoC were equivalent in terms of clinical effectiveness while the six observational studies considered the two MoC as equivalent but only based on previous published studies.

**Fig. 2:**
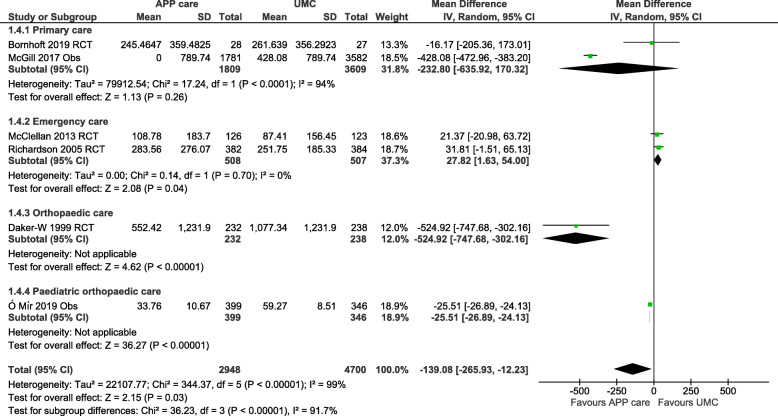
Funnel plots of health care costs per patient for advanced practice physiotherapy care compared to usual medical care in primary care, emergency departments and adult and paediatric orthopaedic care. Costs in euro 2020 (adjusted for inflation & converted). Health care costs measured in included studies: salaries, diagnostic tests, medication prescriptions and follow-up care with a 2 to 12 months time horizon. CI: Confidence intervals; IV: Inverse variance method; Obs: Observational study; RCT: Randomized controlled trial; SD: Standard deviation. McGill, 2017: Only the between-group differences (APP vs UMC) in health care cost were reported in the original study. Ó Mír et al., 2019: UMC comparison group is based on imputed costs

For health system costs, six studies (four RCTs and two cohorts) in primary, emergency, and specialized secondary care (orthopaedic and paediatric) were pooled into a meta-analysis. One of the included studies was of high quality [31], four of moderate [32–34, 40, 41] and one of low quality [36]. Health care system costs per patient were significantly lower with APP care than with UMC (MD: − 139.08 €; 95%CI: − 265.93 to − 12.23; *n* = 7648; I^2^ = 99%; *p* = 0.03) at 2 to 12-month follow-up, as presented in Fig. 2. Among the different clinical settings, health care system costs with APP care were significantly lower in orthopaedic care and paediatric orthopaedic care but significantly higher in emergency care. A secondary analysis of health system costs per patient including only RCTs reported no significant difference between the costs of APP care and UMC (MD: -52.84 € in favor of APP; 95%CI: -153.35 to 47.66; I^2^ = 87%; *n* = 1540; *p* = 0.30) (Fig. S1 in supplementary material). Four observational studies could not be pooled since standard deviations were not available in the original publications or after contacting the authors [35, 37–39]. Mean health system costs were lower with APP care than with UMC in these four studies. When excluding the two trials in emergency care, all included studies reported a lower mean health care system costs per patient with APP care than with UMC (range MD: -12.19 to -524.92 €; 8 studies), as presented in Table 3.

**Table 3:** Health care costs, patient costs and productivity losses methodology and health care costs mean difference between advanced practice physiotherapy care and usual medical care

	Author, year	Follow-up	Economic perspective	Original currency	Health care costs				Patient costs				Productivity losses		Economic analyses			APP-UMC Health care system costs differences per patient (in euro)	
					Salary	Diagnostic tests	Medications	Follow-up care	Travel cost	Prescription cost	Private meals	Private treatment	Work losses	Work compensation	CMA	CUA	CBA		
																		Mean	95% CI
RCTs	Bornhöft et al., 2019	12 months	Societal	Euro 2014-17	✓	✓	✓	✓					✓	✓	✓	✓	✓	-31.9	-361.61 to 297.79
	Daker-White et al., 1999	5.6 ± 1.3 months	Health care & patient	Pound 1996-97	✓	✓	✓	✓	✓	✓	✓	✓			✓			-524.92	-754.03 to -295.76
	McClellan et al., 2013	8 weeks	Health care & patient	Pound 2007-08	✓		✓	✓	✓	✓					✓			21.37	-20.97 to 63.72
	Richardson et al., 2005	6 months	Societal	Pound 2001-02	Unclear^a^				Unclear^a^				Unclear^a^		✓			31.81	-1.40 to 65.02
Observational studies	Belthur et al.,2003	No	Health care	Pound 2003	✓										✓^b^			-12.19	NA
	Brennen et al., 2019	No	Health care	AUD 2016^c^	✓										✓^b^			range: -47.09 to -5.65	
	Cottrell et al., 2019	No	Health care	AUD 2017	✓										✓^b^			Telehealth is 13% (95% CI: 10 to 16%) less expensive than face-to-face	
	Harding et al., 2018	No	Health care & patient^c^	AUD 2014-15^c^	✓				d						✓^b^			-17.94	NA
	McGill, 2017 & McGill et al., 2021	Unclear but ≤18 months	Health care	USD 2016-17^c^	✓	✓	✓	✓							✓^b^			-428.08	-472.96 to -383.21
	Ó Mír et al., 2019	12 months	Health care	Euro 2017	✓			✓^e^							✓^b^			-25.51	-26.89 to -24.12
	Peterson et al., 2021	No	Health care & patient	Euro 2019	✓				d						✓			-34.22	NA
Modeling	Coman et al., 2014 & Standfield et al., 2016	5.2 months^f^	Health care	AUD 2014	✓	✓	✓	✓								✓			

Costs in euro 2020 (adjusted for inflation & converted in euro)

*APP* Advanced practice physiotherapy, *AUD* Australian dollar, *CA* Cost analysis, *CBA* Cost-benefit analysis, *CEA* Cost-effectiveness analysis, *CI* Confidence intervals, *CMA* Cost minimization analysis, *CUA* Cost-utility analysis, *NA* Not available, *RCTs* Randomized controlled trials, *UMC* Usual medical care, *USD* American dollar

^a^In the study by Richardson et al., 2005, costs include health and social costs in the acute hospital and community, personal out of pocket expenses and productivity losses to the society without providing more details

^b^APP clinical effectiveness not directly assessed in the study but demonstrated in previous studies

^c^Exact financial year not confirmed in the article

^d^Include patient wait time in the clinic

^e^Only include salary

^f^Economic model time horizon in Standfield et al., (2016) was 5.25 years

**Fig. 3:**
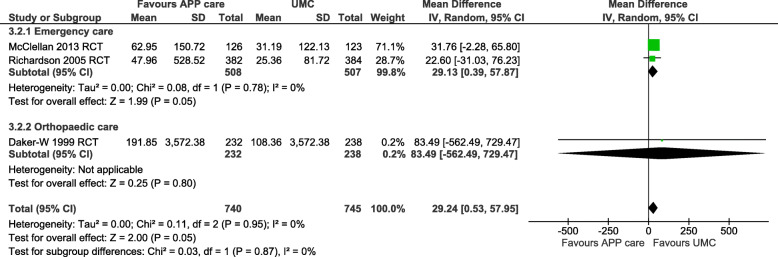
Funnel plots on patient costs per patient for advanced practice physiotherapy care compared to usual medical care in emergency and orthopaedic care. Costs in euro 2020 (adjusted for inflation & converted). Patient costs included: travel costs, waiting time, prescription costs, private meals, and private treatment with a 2 to 6 months time horizon. CI: Confidence intervals; IV: Inverse variance method; RCT: Randomized controlled trial; SD: Standard deviation

For patient costs, two moderate quality RCTs in emergency [33, 34] and one moderate quality RCT in orthopaedic care [32] were pooled into a meta-analysis. Patient costs per patient were significantly higher with APP care than UMC (MD: 29.24 €; 95%CI: 0.53 to 57.95; *n* = 1485; I^2^ = 0%; *p* = 0.05) at 2 to 6-month follow-up, as showed in Fig. 3.

**Fig. 4:**
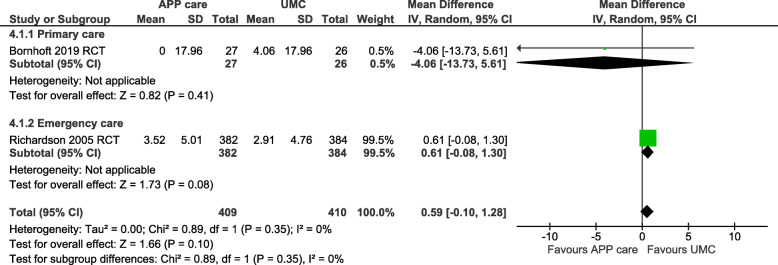
Funnel plots of productivity losses per patient for advanced practice physiotherapy care compared to usual medical care in primary and emergency care. Costs in thousands of euro 2020 (adjusted for inflation & converted). Productivity losses included: work losses and work compensation with a 6 to 12 months time horizon. CI: Confidence intervals; IV: Inverse variance method; RCT: Randomized controlled trial; SD: Standard deviation

For productivity losses, one high quality RCT in primary care [31] and one moderate quality RCT in emergency care [34] were pooled into a meta-analysis. No significant differences between APP MoC and UMC were reported (MD: 590 €/patient in favour of UMC; 95%CI: -100 to 1280; *n* = 819; I^2^ = 0%; *p* = 0.1) at 6 to 12-month follow-up, as presented in Fig. 4.

#### Cost-utility and cost-benefit analyses

The high quality RCT by Bornhöft et al. [31] performed a cost-utility analysis comparing APP care and UMC. The incremental cost-effectiveness ratio (ICER) mean estimation was that APP care was more effective and cost saving than UMC, and that there was only a 7 to 8% chance that UMC was more effective and cost saving. Based on the cost-benefit analysis, there was 85% chance that APP care was cost-effective at a willingness to pay of 20,000 €/QALY.

Based on the modeling study by Coman et al. [43], an APP MoC involving an APP-led multidisciplinary team management (APP, occupational therapist, psychologist, pharmacist, etc.) for non-surgical candidates was more expensive than UMC in orthopaedic care by 72.32 €/patient but resulted in a net incremental benefit of 0.23 QALY. Regarding the cost-utility analysis, the mean incremental cost-effectiveness ratio (ICER) was 319.62 €/QALY, with a credibility interval with a range from a dominant scenario where the multidisciplinary APP MoC was more effective and cost-saving in 37% of cases to 2237.98 €/QALY for the 95% upper limit of the credibility interval. Based on the discrete event simulation with dynamic queuing by Standfield et al. [44], the addition of 100 new patients in the multidisciplinary APP MoC would result in an incremental 0.05 QALYs/patient when compared to UMC. The ICER of adding 100 new patients in the multidisciplinary APP MoC is 5518.1 €/QALY (95%CI: 4830.43 to 6241.92).

#### Economic evaluations of advanced practice physiotherapy care compared to other health care providers care

**Fig. 5:**
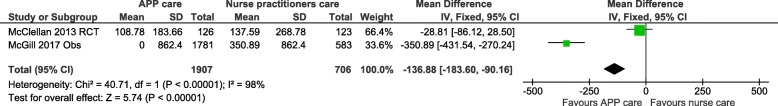
Funnel plots on health care costs per patient in advanced practice physiotherapy care compared to nurse practitioners in primary and emergency care. Costs in euro 2020 (adjusted for inflation & converted in euro). Health care costs measured in included studies: salaries, diagnostic tests, medication prescriptions and follow-up care with a ≥ 2 months time horizon. CI: Confidence intervals; IV: Inverse variance method; Obs: Observational study; RCT: Randomized controlled trial; SD: Standard deviation. McGill, 2017: Only the between-group differences (APP vs UMC) in health care cost were reported in the original study. Exact mean costs per patient for APP and UMC were not reported

Two studies performed cost minimization analyses and compared health system costs with APP care with other health care providers. Two moderate quality studies (one RCT and one cohort study) compared APP care to nurse practitioner care in primary and emergency care were pooled into a meta-analysis [33, 40, 41]. Health care system costs per patient were significantly lower with APP care compared to nurse practitioners care (MD: -136.88 €; 95%CI: -183.6 to -90.16; *n* = 2613; I^2^ = 98%; *p* < 0.0001) as showed in Fig. 5.

Based on the observational study by McGill [40, 41], health care system costs per patient were significantly lower with APP care compared to osteopathic physicians (MD: -245.74 €; 95%CI: -398.19 to -93.29; *n* = 1926; *p* = 0.002) or physician assistant care (MD: -384.37 €; 95%CI: -435.27 to -333.47; *n* = 3743; *p* < 0.0001).

#### Advanced practice physiotherapy telehealth care compared to face-to-face care

Based on one low quality observational study in orthopaedic care, telehealth APP care is 13% (95%CI: 10 to 16%; *n* = 44) less expensive than face-to-face APP care with no increase in adverse events reported. The authors concluded that telehealth APP care is a viable option, especially for individuals living in rural areas [42].

### GRADE approach level of evidence

Pooled results comparing APP care to UMC were considered of low quality for health system costs and for patient costs and of very low quality for productivity losses results. Pooled result for health care system costs comparing APP care to nurse practitioners care was considered of low quality. See details of Grade Approach and conclusions in Table 4.

**Table 4:** Summary of findings from meta-analyses and GRADE analyses of the evidence on health care costs, patient costs and productivity losses

Economic perspective	Clinical setting	Main results (95%CI) APP care compared to UMC	No. of participants (RCTs & Obs)	Quality of included studies based on EPHPP (no. of studies)	Certainty (GRADE)	Conclusions
Health care costs	Primary, emergency, orthopaedic & paediatric care	Costs per patient were **139.08 € lower** (12.23 to 265.93) with APP care	7648 (4 RCTs & 2 Obs)	Strong: 1 Moderate: 4 Weak: 1	Low (1, 2, 4, 5)	Evidence suggests that health care costs per patient are lower with APP care than UMC. Costs difference is large but uncertain, as cost is higher with APP care in emergency care.
Patient costs	Emergency & orthopaedic care	Costs per patient were **29.24 € higher** (0.53 to 57.95) with APP care	1485 (3 RCTs)	Moderate: 3	Low (2, 3)	Evidence suggests that patient costs per patient are significantly higher with APP care compared to UMC. Costs difference is small.
Productivity losses	Emergency & orthopaedic care	Costs per patient were 590 € higher (− 100 to 1280) with APP care	819 (2 RCTs)	Strong: 1 Moderate: 1	Very low (2, 3, 4)	Evidence is very uncertain
Secondary analysis		APP care compared to nurse practitioners care				
Health care costs	Primary & emergency care	Costs per patient were **136.88 € lower** (90.16 to 183.6) with APP care	2613 (1 RCT & 1 Obs)	Moderate: 2	Low (1, 2, 4, 5)	Evidence suggests that health care costs per patient is lower with APP care than nurse practitioners care

Results in **bold are statistically significant**

1. Initially rated as moderate (some information from observational studies)

2. Downgraded due to risk of bias (most information is from studies at moderate risk of bias)

3. Downgraded due to imprecision of the results

4. Downgraded due to inconsistency of the results

5. Upgraded due to large effect of the results

Health care costs measured in included studies: salaries, diagnostic tests, medication prescriptions and follow-up care with a 2 to 12 months time horizon

Patient costs included: travel costs, waiting time, prescription costs, private meals, and private treatment with a 2 to 6 months time horizon

Productivity losses included: work losses and work compensation with a 6 to 12 months time horizon

GRADE Working Group grades of evidence:

**High quality**: We are very confident that the true effect lies close to that of the estimate of the effect

**Moderate quality**: We are moderately confident in the effect estimate: The true effect is likely to be close to the estimate of the effect, but there is a possibility that it is substantially different

**Low quality**: Our confidence in the effect estimate is limited: The true effect may be substantially different from the estimate of the effect

**Very low quality**: We have very little confidence in the effect estimate: The true effect is likely to be substantially different from the estimate of effect

*€* euro, *APP* Advanced practice physiotherapy, *CI* Confidence interval, *EPHPP* Effective Public Health Practice Project, *Obs* Observational studies, *RCT* Randomized controlled trial, *UMC* Usual medical care

## Discussion

### Main findings

This systematic review synthesizes, for the first time, evidence on the economic evaluations of various APP MoC. Four RCTs, seven analytical cohort studies and one modeling economic study were included. Most of the included participants in these MoC were adults with MSKDs, although infants and children with MSKDs and women with pelvic floor disorders were intended populations in some models. Overall, low quality evidence suggests that APP MoC lead to lower health care system cost but higher patient cost per patient when compared to UMC.

### Strength and limitations

Strengths of this review include systematic searches of four important bibliographical databases and the use of the validated Effective Public Health Practice Project tool to assess global methodological quality and of the CASP checklist to assess the quality of the included studies and their economic evaluations. The use of GRADE also represents a strength of our review, as it allowed a more objective and standardized analysis of the quality of evidence. Our review also included studies in different clinical setting and countries therefore increasing the generalizability of the results.

However, some limitations need to be highlighted in the interpretation of our results. First, this review is mainly limited by the quality of the available evidence as only one high quality study was included. Although the inclusion of studies from different clinical settings and countries could be seen as a strength, it also led to heterogeneity among the results. Various methodologies were used to estimate health care cost, including slightly different time horizons, which also led to heterogeneity among the results. The presented meta-analyses, and especially the sub-group analyses, are also based on a relatively small number of studies; which could inaccurately estimate the between-study variance, and therefore the precision of the estimates [45]. Many observational studies could not be pooled in meta-analyses as standard deviation from raw results were not available.

### Interpretation and implication of the results

Our meta-analysis suggests that health care system costs per patient are lower with APP MoC than with UMC and the cost saving might be considered large. However, measured costs differences were inconsistent among studies. For example, the sensitivity analysis including only RCTs did not show significant differences in costs between MoC. Furthermore, the subgroup analysis including only the two-emergency care RCTs concluded that APP care is more expensive than UMC, albeit only slightly. This suggests that the costs saving with APP care may be dependent on the clinical setting. However, very large cost saving with APP care were reported in the two other studies that evaluated APP MoC in primary care and in an orthopaedic specialized setting. In the RCT by Daker-White et al. [32], APPs referred 2.4 times fewer patients for surgery while being as effective as the usual MoC in terms of reduction of patients’ pain and disability and resulted in a mean cost saving of 473.04 €/patient. In the American observational study by McGill [40, 41], the important cost saving with APP care was largely due to a reduction in physicians’ salary and the number of medication prescriptions, leading to a difference in costs of 427.25 €/patient. This large difference could be explained by higher medication expenses per capita [46] and medical doctors’ salaries [47–49] found in the United States and not found in other studies that are mainly from Europe in this review. Overall, our results suggest that in certain circumstances APP care has the potential to generate very important saving to health care systems when compared to UMC. It is also important to point out that observational studies (*n* = 4) not included in these meta-analyses also systematically reported lower health care costs with APP care (5.65 to 47.09 €/patient), although these observational studies only included health care provider salaries as health care costs. In sum, our results do suggest that health care costs per patient are lower with APP care but uncertainties remain and this is likely dependent on several factors such as: in which health care system the MoC is being assessed, reimbursement schemes of physicians and professionals, the MoC clinical setting (as mentioned earlier) and the population being cared for. The exact role of APPs and the types of evaluation and treatments offered are to be considered also as they involve the use of various health care resources such as prescription of imaging tests, medications, injections and referral to rehabilitation or to surgery.

Another finding of our review is that APP care was slightly more expensive from the patient’s perspective, but these results should also be interpreted cautiously and have been appraised as being of low-quality evidence in our GRADE appraisal. The cost differences observed are small and they appear to be driven by the two-emergency care RCTs included in our review. In the RCT by Richardson et al. [34], mean patients costs were higher in the APP group, but were highly skewed and influence by extreme values, as mentioned by the authors. In the RCT by Daker-White et al. [32], the mean cost difference was largely attributable to a single patient enrolled in the APP group that opted to pay for a private spine surgery (total cost: 14,397 €).

Results regarding productivity losses should also be interpreted cautiously as they are based on very low quality evidence. The confidence interval of the productivity losses obtained in our review is large and not statistically significant. High uncertainty in the difference in productivity losses highlight the need for further research. Several authors report that MSKDs often lead to significant productivity losses among workers [50–52]. Therefore, as APP care may facilitate earlier return to work by decreasing wait time for an initial consultation or by providing better rehabilitation care [12, 19, 32, 53, 54], the cost saving could be important, but this as yet to be formally confirmed in future studies on APP.

As discussed above, most of the included studies did not measure costs using a societal perspective limiting our conclusion mainly to the health care system perspective. Also, the methodology to estimate costs varied among the included studies, with some studies only including APP, doctors and administrative staff salaries as health care costs and did not consider other costs such as diagnostic tests or medications.

Our results also suggest that health care system costs are lower with APP care than with other allied health care providers such as nurse practitioners when substituting physicians in new models of care aimed at improving access to care while delivering efficient care. A previous meta-analysis specific to nurse practitioner MoC, reported lower health care cost with nurse practitioner care compared to UMC in primary care [55]. However, the reported cost saving with nurse practitioner care was small (MD: − 6.41 € 2006; 95%CI: − 9.28 to − 3.55; 2 RCTs, *n* = 2689). Based on our results and on previous systematic reviews, APP care could be a more cost-effective alternative to usual medical care than care provided by other allied health care providers such as nurse practitioners.

### Comparison with previous systematic reviews

This systematic review is the first to perform a thorough review of all available evidence on the economic evaluation of APP MoC and to perform meta-analyses on health care and patients costs as well as productivity losses compared to UMC. Previous systematic reviews could not conclude on the economic benefits of APP MoC. Indeed, these reviews reported that APP MoC improve health care access while providing at least comparable quality of care than UMC [12–17, 19]. As our results suggest health care system cost saving with APP MoC compared to UMC, the development and implementation of these models is further supported, especially for MSKDs care.

### Unanswered questions and future research

As our results are based on low to very low quality evidence, the true cost differences between APP MoC and UMC might be markedly different from the estimated costs differences, especially for productivity losses, in which our results are very uncertain. The economic evaluation of MoC is also context dependent and our results might not be generalizable to different countries, especially for non-Western Countries. Therefore, high-quality studies regarding the economic impact of APP MoC in different countries and setting are still needed. These studies should be carefully designed to minimize the potential bias and capture all cost components related to APP MoC. Future studies should also conduct cost-effectiveness, cost-utility and/or cost-benefit analyses, as these analyses provide more complete and meaningful data.

## Conclusions

Low quality evidence suggests that health care system costs per patient are lower with APP care than UMC. The overall cost saving may be large but appear inconsistent among studies. Low quality evidence suggests that patient costs were higher with APP care than UMC, although the observed cost difference was small. Regarding productivity losses, the current level of evidence is very uncertain. Costs differences may vary depending on various factors such as the cost methodology used and on the clinical setting. More evidence is needed to fully evaluate cost benefits of APP models of care.

## Supplementary Information

**Additional file 1.**

**Additional file 2.**

**Additional file 3.**

## Data Availability

The Excel dataset is available in supplementary materials, for further details, contact the corresponding author (SL).

## Abbreviations

APP: Advanced practice physiotherapy
APPs: Advanced practice physiotherapists
AUD: Australian dollar
CASP: Critical Appraisal Skills Programme
CBA: Cost-benefit analysis
CEA: Cost-effectiveness analysis
CI: Confidence intervals
CMA: Cost minimization analysis
CUA: Cost-utility analysis
CT: Computerized tomography
EMG: Electromyography
EPHPP: Effective Public Health Practice Project
GRADE: Grading of Recommendations, Assessment, Development and Evaluations
ICER: Incremental cost-effectiveness ratio
IV: Inverse variance method
MD: Mean differences
MoC: Models of care
MRI: Magnetic resonance imaging
MSK: Musculoskeletal
MSKDs: Musculoskeletal disorders
NA: Not available
Obs: Observational studies
OECD: Organisation for Economic Co-operation and Development
QALYs: Quality-adjusted life years
RCTs: Randomized controlled trials
SD: Standard deviation
UMC: Usual medical care
USD: American dollar
UK: United Kingdom
X-ray: Radiograph
