# Correction to: Economic evaluation of advanced practice physiotherapy models of care: a systematic review with meta-analyses

**DOI:** 10.1186/s12913-021-07406-z

**Published:** 2022-03-10

**Authors:** Simon Lafrance, Anthony Demont, Kednapa Thavorn, Julio Fernandes, Carlo Santaguida, François Desmeules

**Affiliations:** 1 School of Rehabilitation, Faculty of Medicine, Université de Montréal, Montreal, Quebec Canada; 2 Maisonneuve-Rosemont Hospital Research Center, Université de Montréal Affiliated Research Center, Montreal, Quebec Canada; 3 INSERM 1123 ECEVE, Faculty of Medicine, Paris-Diderot University, Paris, France; 4 Physiotherapy School, University of Orleans, Orleans, France; 5 School of Epidemiology and Public Health, University of Ottawa, Ottawa, ON Canada; 6 Ottawa Hospital Research Institute, Ottawa, ON Canada; 7 Hôpital du Sacré-Coeur de Montréal Research Center, Université de Montréal Affiliated Research Center, Montreal, Quebec Canada; 8 Department of Surgery, Faculty of Medecine, Université de Montréal, Montreal, Quebec Canada; 9 Department of Neurology and Neurosurgery, Faculty of Medecine, McGill University Health Center, Montreal, Quebec Canada

**Correction to: BMC Health Serv Res 21, 1214 (2021)**

**https://doi.org/10.1186/s12913-021-07221-6**

Following publication of the original article [1], errors in the conversion rates of cost into Euros were discovered. All cost calculations were corrected and all meta-analyses were recalculated. Overall, these errors only slightly modified the cost estimates. The significance of the results and original conclusions of the review did not change following these corrections.

The corrections in the main text are indicated in the Additional file 1 and implemented in the original article. In addition, Figs. 2, 3, 4 and 5 and Tables 3 and 4 have been updated in the original article.

## Supplementary Information

**Additional file 1.**
