# Supplementary material for: Economic evaluation of advanced practice physiotherapy models of care: a systematic review with meta-analyses
Source: BMC Health Serv Res. 2021 Nov 9;21:1214. doi: 10.1186/s12913-021-07221-6 (PMC8579553; doi:10.1186/s12913-021-07221-6)
Supplement: Supplementary file 1 — Additional file 1. [file 12913_2021_7221_MOESM1_ESM.docx]

MEDLINE, Embase, Cochrane

All Ovid MEDLINE(R) 1946 to Present:

Embase 1974 to Present

EBM Reviews - Cochrane Central Register of Controlled Trials

1. Physical Therapy Specialty/
2. Physician Assistants/
3. Triage/
4. Specialization/
5. Emergency Service, Hospital/
6. advance* practice*.ti,ab,kw.
7. ((enhan* or expan* or exten*) adj2 (scope* or practice*)).ti,ab,kw.
8. (role* adj2 (enhan* or expan* or exten*)).ti,ab,kw.
9. (physician* adj2 (exten* or assist*)).ti,ab,kw.
10. (advance* practice* or primary contact* or specialist* or triage*).ti,ab,kw.
11. (emergency adj2 (department* or room* or service* or ward*)).ti,ab,kw.
12. (trauma adj2 (center* or centre*)).ti,ab,kw.
13. 1 or 2 or 3 or 4 or 5 or 6 or 7 or 8 or 9 or 10 or 11 or 12
14. Physical Therapists/
15. kinesitherap*.ti,ab,kw.
16. physical therap*.ti,ab,kw.
17. physiotherap*.ti,ab,kw.
18. 14 or 15 or 16 or 17
19. "Costs and Cost Analysis"/
20. Cost-Benefit Analysis/
21. Quality-Adjusted Life Years/
22. Health Care Costs/
23. "cost of illness"/
24. Health expenditures/
25. Economics/
26. (cost* adj2 (analys* or benefit* or effectiv* or utilit* or care)).ti,ab,kw.
27. (cost* adj2 (illness or disease* or sickness)).ti,ab,kw.
28. (burden* adj2 economic*).ti,ab,kw.
29. (economic* adj2 analys*).ab,kw,ti.
30. (quality-adjusted life year* or quality adjusted life year* or QALY*).ab,kw,ti.
31. (out-of-pocket adj2 (payment* or expenditure* or cost* or spending or expense*)).ab,kw,ti.
32. ((adjusted or quality-adjusted) adj2 year*).ab,kw,ti.
33. 19 or 20 or 21 or 22 or 23 or 24 or 25
34. 13 and 18 and 33
35. 34 not (exp animals/ not humans.sh.)

CINAHL:

MH ("Physician Assistants" OR "Emergency Service") OR TI ("advance* practice*" OR "enhan* N2 scope*" OR "enhan* N2 practice*" OR "expan* N2 scope*" OR "expan* N2 practice*" OR "exten* N2 scope*" OR "exten* N2 practice*" OR "role* N2 enhan*" OR "role* N2 expan*" OR " role* N2 ext*" OR " physician* N2 exten*" OR " primary contact*" OR " specialist*" OR " triage*" OR AB ("advance* practice*" OR "enhan* N2 scope*" OR "enhan* N2 practice*" OR "expan* N2 scope*" OR "expan* N2 practice*" OR "ext* N2 scope*" OR "exten* N2 practice*" OR "role* N2 enhan*" OR "role* N2 expan*" OR " role* N2 exten*" OR " physician* N2 exten*" OR " primary contact*" OR " specialist*" OR " triage*" OR "emergency N2 department" OR "emergency N2 room" OR "emergency N2 service" OR "emergency N2 ward" OR "trauma N2 center")

AND

MH ("Physical Therapist" OR "Physiotherapy") OR TI ("kinesiotherap*" OR "physical therap*" OR "physio*") OR AB ("kinesiotherap*" OR "physical therap*" OR "physio*")

AND

MH ("Cost and Cost Analysis" OR "Cost-Benefit Analysis" OR "Quality-Adjusted Life Years" OR "Health Care Costs" OR "Economic Aspects of Illness" OR "Economics") OR TI ("cost* N2 analys*" OR "cost* N2 benefit" OR "cost* N2 effectiv*" OR "cost* N2 utilit*" OR "Quality-Adjusted Life Year*" OR" Quality Adjusted Life Year*" OR "quality-adjusted N2 year**" OR "adjusted N2 year**" OR "QALY*" OR "care N2 cost*" OR "cost* N2 illness" OR "cost* N2 disease" OR "cost* N2 sickness" OR "burden* N2 economic*" OR "economic* N2 analys*" OR "out-of-pocket N2 payment" OR "out-of-pocket N2 expenditure*" OR "out-of-pocket N2 cost*" OR "out-of-pocket N2 spending" OR "out-of-pocket N2 expense*" O N2 expenditure*") OR AB ("cost* N2 analys*" OR "cost* N2 benefit" OR "cost* N2 effectiv*" OR "cost* N2 utilit*" OR "Quality-Adjusted Life Year*" OR "QALY*" OR "care N2 cost*")
