# Supplementary material for: Economic evaluation of advanced practice physiotherapy models of care: a systematic review with meta-analyses
Source: BMC Health Serv Res. 2021 Nov 9;21:1214. doi: 10.1186/s12913-021-07221-6 (PMC8579553; doi:10.1186/s12913-021-07221-6)
Supplement: Supplementary file 2 — Additional file 2. [file 12913_2021_7221_MOESM2_ESM.docx]

# Inflation rates

|  | **AVERAGE YEARLY INFLATION** | | | | | |
| --- | --- | --- | --- | --- | --- | --- |
| YEAR | CAN | EUROPE | UK | AUS | USA | SWE |
| 2020 | 136.45 | *106.06* | 292.03 | 116.47 | 257.45 | 334.31 |
| 2019 | 135.98 | *105.42* | 288.80 | 114.78 | 255.65 | 334.26 |
| 2018 | 133.38 | *103.89* | 281.58 | 112.97 | 251.10 | 328.40 |
| 2017 | 130.43 | *101.96* | 272.48 | 110.83 | 245.12 | 322.11 |
| 2016 | 128.38 | *100.25* | 263.05 | 108.78 | 239.99 | 316.43 |
| 2015 | 126.57 | *100.00* | 258.54 | 107.38 | 237.00 | 313.35 |
| 2014 | 125.16 | *99.90* | 256.03 | 105.78 | 236.72 | 313.49 |
| 2013 | 122.82 | *99.35* | 250.11 | 103.03 | 232.95 | 314.06 |
| 2012 | 121.68 | *97.88* | 242.73 | 100.66 | 229.59 | 314.20 |
| 2011 | 119.86 | *95.36* | 235.18 | 98.79 | 224.92 | 311.43 |
| 2010 | 116.47 | *92.49* | 223.56 | 95.67 | 218.08 | 303.46 |
| 2009 | 114.43 | *90.60* | 213.68 | 93.06 | 214.56 | 299.66 |
| 2008 | 114.09 | *89.72* | 214.83 | 91.20 | 215.25 | 300.61 |
| 2007 | 111.45 | *86.55* | 206.58 | 87.51 | 207.34 | 290.51 |
| 2006 | 109.12 | *84.58* | 198.11 | 85.46 | 201.56 | 284.22 |
| 2005 | 106.98 | *82.75* | 191.98 | 82.59 | 195.27 | 280.41 |
| 2004 | 104.66 | *81.00* | 186.69 | 80.47 | 188.91 | 279.15 |
| 2003 | 102.75 | *79.40* | 181.32 | 78.63 | 184.00 | 278.10 |
| 2002 | 99.99 | *77.87* | 176.18 | 76.48 | 179.87 | 272.84 |
| 2001 | 97.78 | *76.29* | 173.35 | 74.24 | 177.04 | 267.08 |
| 2000 | 95.38 | *74.65* | 170.25 | 70.81 | 172.19 | 262.00 |
| 1999 | 92.85 | *73.26* | 165.41 | 68.21 | 166.58 |  |
| 1998 | 91.27 | *72.40* | 162.88 | 67.26 | 163.01 |  |
| 1997 | 90.37 | *71.48* | 157.49 | 66.88 | 160.53 |  |
| 1996 | 88.93 | *70.26* | 152.71 | 66.53 | 156.86 |  |
| 1995 | 87.55 |  | 149.06 | 64.80 | 152.38 |  |

# Conversion rates summary

|  | **AVERAGE YEARLY RATE SUMMARY** | | |
| --- | --- | --- | --- |
| YEAR | **USD to EUR** | **GBP to EUR** | **AUD to EUR** |
| 2020 | 0.91 | 1.15 | 0.59 |

1 USD = 0.91 euro (€); 1 Pound Sterling (£) = 1.15 euro (€); 1 AUD = 0.59 euro (€)

# Conversion rates details

|  | **End of the month conversion rates and average conversion rate calculation details** | | | | | | | | | |
| --- | --- | --- | --- | --- | --- | --- | --- | --- | --- | --- |
|  | GBP to AUD | GBP to CAD | GBP to USD | **GBP to EUR** | AUD to GBP | **AUD to EUR** | CAD to GBP | **CAD to EUR** | USD to GBP | **USD to EUR** |
| 2020-01-31 | 1.97 | 1.74 | 1.32 | **1.19** | 0.51 | **0.60** | 0.57 | **0.68** | 0.76 | **0.90** |
| 2020-02-29 | 1.98 | 1.71 | 1.28 | **1.16** | 0.51 | **0.59** | 0.58 | **0.68** | 0.78 | **0.91** |
| 2020-03-31 | 2.03 | 1.77 | 1.24 | **1.13** | 0.49 | **0.56** | 0.57 | **0.64** | 0.81 | **0.91** |
| 2020-04-30 | 1.93 | 1.75 | 1.26 | **1.15** | 0.52 | **0.60** | 0.57 | **0.66** | 0.79 | **0.91** |
| 2020-05-31 | 1.86 | 1.71 | 1.24 | **1.11** | 0.54 | **0.60** | 0.59 | **0.65** | 0.81 | **0.90** |
| 2020-06-30 | 1.80 | 1.68 | 1.24 | **1.10** | 0.56 | **0.61** | 0.59 | **0.65** | 0.81 | **0.89** |
| 2020 average | 1.93 | 1.73 | 1.26 | **1.15** | 0.52 | **0.59** | 0.58 | **0.66** | 0.79 | **0.91** |

2020 average was calculated for the first 6-months as analyses were performed in July 2020

1 Pound Sterling (£) = 1.93 AUD; 1 Pound Sterling (£) = 1.73 CAD; 1 Pound Sterling (£) = 1.26 USD; 1 Pound Sterling (£) = 1.15 euro (€); 1 AUD = 0.52 Pound Sterling (£); 1 AUD = 0.59 euro (€); 1 CAD = 0.58 Pound Sterling (£); 1 CAD = 0.66 euro (€); 1 USD = 0.79 Pound Sterling (£) and 1 USD = 0.91 euro (€).

# Inflation and conversion for each study

|  |  |  | Inflation | Conversion | **Inf & Con** |
| --- | --- | --- | --- | --- | --- |
|  |  |  | 2020 | euro | **euro 2020** |
| Bornhöft et al., 2019 | Euro | 2014/2017 | 1.0635542 | 1 | **1.0635542** |
| Daker-White et al., 1999 | Pound | 1996/1997 | 1.88287127 | 1.15 | **2.1616726** |
| McClellan et al., 2013 | Pound | 2007/2008 | 1.38601487 | 1.15 | **1.59124547** |
| Richardson et al., 2005 | Pound | 2001/2002 | 1.67098989 | 1.15 | **1.91841744** |
| Belthur et al.,2003 | Pound | 2003 | 1.61062598 | 1.15 | **1.84911529** |
| Brennen et al., 2019 | AUD | 2016 | 1.07062969 | 0.59 | **0.62783735** |
| Harding et al., 2018 | AUD | 2014/2015 | 1.09281414 | 0.59 | **0.64084673** |
| McGill, 2017 | USD | 2016/2017 | 1.06141247 | 0.91 | **0.9615304** |
| Ó Mír et al., 2019 | Euro | 2017 | 1.04019419 | 1 | **1.04019419** |
| Coman et al., 2014 | AUD | 2014 | 1.10107934 | 0.59 | **0.64569359** |
| Peterson et al., 2021 | Euro | 2019 | 1.00014958 | 1 | **1.00014958** |
| Standfield et al., 2016 | AUD | 2014 | 1.10107934 | 0.59 | **0.64569359** |
