# Supplementary material for: Economic evaluation of advanced practice physiotherapy models of care: a systematic review with meta-analyses
Source: BMC Health Serv Res. 2021 Nov 9;21:1214. doi: 10.1186/s12913-021-07221-6 (PMC8579553; doi:10.1186/s12913-021-07221-6)
Supplement: Supplementary file 3 — Additional file 3. [file 12913_2021_7221_MOESM3_ESM.docx]

**Supplementary material 1**

**FIGURE S1.** Sensitivity analysis: funnel plots of health care cost per patient for advanced practice physiotherapy care compared to usual medical care in primary, emergency, adult and paediatric orthopaedic care in randomized controlled trials.


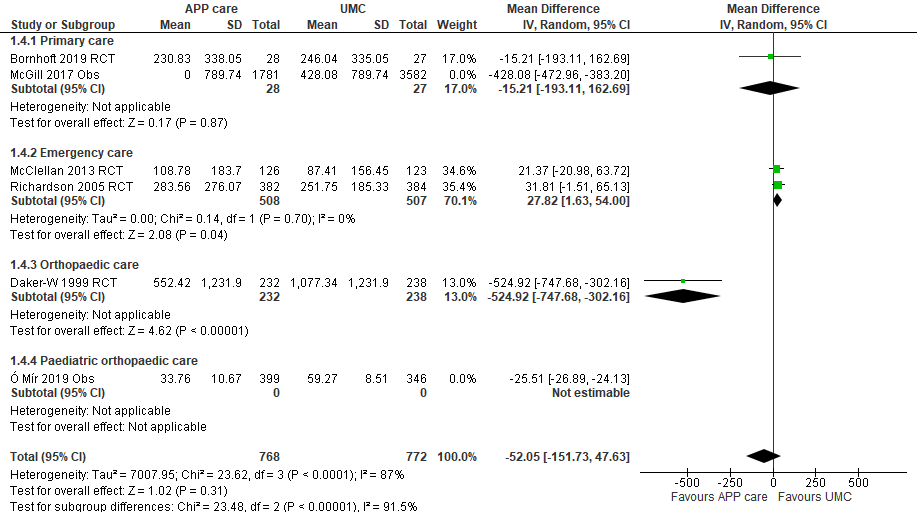


Costs in euro 2020 (adjusted for inflation & converted)

Health care costs measured in included studies: salaries, diagnostic tests, medication prescriptions and follow-up care with a 2 to 12 months time horizon.CI: Confidence intervals; IV: Inverse variance method; Obs: Observational study; RCT: Randomized controlled trial; SD: Standard deviation.

# Table S1. Health care costs, patient costs and productivity losses methodology and health care costs mean difference between advanced practice physiotherapy care and usual medical care. Cost in the original currency.

|  | Author, year | Follow-up | Economic perspective | Original currency | **Health care costs** | | | | **Patient costs** | | | | **Productivity losses** | | **Economic analyses** | | | **APP-UMC Health care costs differences per patient** | |  |  |  |  |
| --- | --- | --- | --- | --- | --- | --- | --- | --- | --- | --- | --- | --- | --- | --- | --- | --- | --- | --- | --- | --- | --- | --- | --- |
|  |  |  |  |  | Salary | Diagnostic tests | Medications | Follow-up care | Travel cost | Prescription cost | Private meals | Private treatment | Work losses | Work compensation | CA | CUA | CBA |  |  |  |  |  |  |
|  |  |  |  |  |  |  |  |  |  |  |  |  |  |  |  |  |  | Mean | 95% CI |  |  |  |  |
| RCTs | Bornhöft et al., 2019 | 12 months | Societal | Euro 2014-16 | ✓ | ✓ | ✓ | ✓ |  |  |  |  | ✓ | ✓ | ✓ | ✓ | ✓ | -30.00 | -340 to 280 |  |  |  |  |
|  | Daker-White et al., 1999 | 5.6±1.3 months | Health care & patient | Pound 1996-97 | ✓ | ✓ | ✓ | ✓ | ✓ | ✓ | ✓ | ✓ |  |  | ✓ |  |  | -242.83 | -348.82 to -136.82 |  |  |  |  |
|  | McClellan et al., 2013 | 8 weeks | Health care & patient | Pound 2007-08 | ✓ |  | ✓ | ✓ | ✓ | ✓ |  |  |  |  | ✓ |  |  | 13.43 | -13.18 to 40.04 |  |  |  |  |
|  | Richardson et al., 2005 | 6 months | Societal | Pound 2001-02 | Unclear † | | | | Unclear † | | | | Unclear † | | ✓ |  |  | 16.58 | -0.73 to -33.89 |  |  |  |  |
| Observational studies | Belthur et al.,2003 | No | Health care | Pound 2003 | ✓ |  |  |  |  |  |  |  |  |  | ✓‡ |  |  | -6.59 | NA |  |  |  |  |
|  | Brennen et al., 2019 | No | Health care | AUD 2016‡ | ✓ |  |  |  |  |  |  |  |  |  | ✓‡ |  |  | range: -75.65 to -10.18 | |  |  |  |  |
|  | Cottrell et al., 2019 | No | Health care | AUD 2017 | ✓ |  |  |  |  |  |  |  |  |  | ✓‡ |  |  | Telehealth is 13% (95% CI: 10 to 16%) less expensive than face-to-face | |  |  |  |  |
|  | Harding et al., 2018 | No | Health care & patient§ | AUD 2014-15‡ | ✓ |  |  |  | ¶ |  |  |  |  |  | ✓‡ |  |  | -28 | NA |  |  |  |  |
|  | McGill, 2017 & McGill et al., 2021 | Unclear but ≤ 18 months | Health care | USD 2016-17‡ | ✓ | ✓ | ✓ | ✓ |  |  |  |  |  |  | ✓‡ |  |  | -445.21 | -491.89 to -398.54 |  |  |  |  |
|  | Ó Mír et al., 2019 | 12 months | Health care | Euro 2017 | ✓ |  |  | ✓ ¶ ¶ |  |  |  |  |  |  | ✓‡ |  |  | -24.52 | -25.85 to -23.19 |  |  |  |  |
|  | Peterson et al., 2021 | No | Health care & patient | Euro 2019 | ✓ |  |  |  | ¶ |  |  |  |  |  | ✓ |  |  | -53 | NA |  |  |  |  |
| Modelling | Coman et al., 2014 & Standfield et al., 2016 | 5.2 months | Health care | AUD 2014 | ✓ | ✓ | ✓ | ✓ |  |  |  |  |  |  |  | ✓ |  |  |  |  |  |  |  |

Costs in euro 2020 (adjusted for inflation & converted in euro).

APP: Advanced practice physiotherapy; AUD: Australian dollar; CA: Cost analysis; CBA: Cost-benefit analysis; CEA: Cost-effectiveness analysis; CI: Confidence intervals; CMA: Cost minimization analysis; CUA: Cost-utility analysis; NA: Not available; RCTs: Randomized controlled trials; UMC: Usual medical care; USD: American dollar

† In the study by Richardson et al., 2005, costs include health and social costs in the acute hospital and community, personal out of pocket expenses and productivity losses to the society without providing more details

‡ APP clinical effectiveness not directly assessed in the study but demonstrated in previous studies

§ Exact financial year not confirmed in the article

¶ Include patient wait time in the clinic

¶¶ Only include salary

¶¶¶ Economic model time horizon in Standfield et al., (2016) was 5.25 years

# Table S2. Baseline pain, disability and quality of life of participants in randomized controlled trials.

| Author, year | Pain (0-100)  ± SD (95%CI) | Disability (0-100) | Quality of life  (0-100) |
| --- | --- | --- | --- |
|  |  |  |  |
|  |  |  |  |
| Bornhöft et al., 2019 | NPRS current pain / mean pain  APP: 71 ± 24 / 57 ± 24  UMC: 66 ± 24 / 48 ± 29 | Disability rating index  APP: 38.6 ± 20.1  UMC: 39.5 ± 25.5 | EQ-5D / VAS  APP: 50 ± 32 / 57.5 ± 20.8  UMC: 59 ± 25 / 63.2 ± 25.7 |
| Daker-White et al., 1999 | VAS overall pain in the last week  APP: 53.5 ± 26.4  UMC: 52.8 ± 25.3 | ODI / WOMAC  APP: 38.8 ± 17.7 / 34.9 ± 21.7  UMC: 38.2 ± 15.5 / 37.4 ± 22.4 | Perceived handicap functional / social activities  APP: 78 ± 30 / 66 ± 37  UMC: 75 ± 34 / 68 ± 38 |
| McClellan et al., 2013 | VAS  APP: 66.7 (59.3 to 74.0)  UMC: 64.2 (56.8 to 71.5)  NP: 60.3 (52.8 to 67.7) | NA | NA |
| Richardson et al., 2005 | APP: 55.4 ± 21.9  UMC: 56.1 ± 22.6 | HAQ disability index  APP: 9.67 ± 18  UMC: 7.33 ± 14.33 | EQ-5D  APP: 45 ± 30  UMC: 47 ± 30 |

Pain: 0=less pain, 100=more pain; Disability: 0=less disability, 100=more disability; Quality of life: 0=less quality of life, 100=more quality of life

All scores were converted on a 0-100 scale.

APP: Advanced Practice physiotherapy; HAQ: Health Assessment Questionnaire; NA: not available; NP: Nurse Practitioners; NPRS: Numerical Pain Rating Scale; ODI: Oswestry Disability Index; UMC: Usual Medical Care; VAS: Visual Analog Scale; WOMAC: Western Ontario and McMaster Universities Osteoarthritis Index
